# Polymyxin P is the active principle in suppressing phytopathogenic *Erwinia spp.* by the biocontrol rhizobacterium *Paenibacillus polymyxa* M-1

**DOI:** 10.1186/1471-2180-13-137

**Published:** 2013-06-18

**Authors:** Ben Niu, Joachim Vater, Christian Rueckert, Jochen Blom, Maik Lehmann, Jin-Jiang Ru, Xiao-Hua Chen, Qi Wang, Rainer Borriss

**Affiliations:** 1 The MOA Key Laboratory of Plant Pathology, Department of Plant Pathology, College of Agronomy and Biotechnology, China Agricultural University, Beijing 100193, PR China; 2 Institut für Biologie/Bakteriengenetik, Humboldt Universität Berlin, Berlin 10115, Germany; 3 Institut für Chemie, Technische Universität Berlin, Berlin 10623, Germany; 4 Computational Genomics, Center for Biotechnology (CeBiTec) Universität Bielefeld, Bielefeld D-33594, Germany; 5 Institut für Biologie/Molekulare Parasitologie, Humboldt Universität Berlin, 10115 Berlin, Germany; 6 ABiTEP GmbH, 12489 Berlin, Germany; 7 Present address: Department of Microbiology and Immunobiology, Harvard Medical School, Boston, MA 02115, USA

## Abstract

**Background:**

Nine gene clusters dedicated to nonribosomal synthesis of secondary metabolites with possible antimicrobial action, including polymyxin and fusaricidin, were detected within the whole genome sequence of the plant growth-promoting rhizobacterium (PGPR) *Paenibacillus polymyxa* M-1. To survey the antimicrobial compounds expressed by M-1 we analyzed the active principle suppressing phytopathogenic *Erwinia spp.*

**Results:**

*P. polymyxa* M-1 suppressed the growth of phytopathogenic *Erwinia amylovora* Ea 273, and *E. carotovora*, the causative agents of fire blight and soft rot, respectively. By MALDI-TOF mass spectrometry and reversed-phase high-performance liquid chromatography (RP-HPLC), two antibacterial compounds bearing molecular masses of 1190.9 Da and 1176.9 Da were detected as being the two components of polymyxin P, polymyxin P_1_ and P_2_, respectively. The active principle acting against the two *Erwinia* strains was isolated from TLC plates and identified by postsource decay (PSD)-MALDI-TOF mass spectrometry as polymyxin P_1_ and polymyxin P_2_. These findings were corroborated by domain structure analysis of the polymyxin (*pmx*) gene cluster detected in the M-1 chromosome which revealed that corresponding to the chemical structure of polymyxin P, the gene cluster is encoding D-Phe in position 6 and L-Thr in position 7.

**Conclusions:**

Identical morphological changes in the cell wall of the bacterial phytopathogens treated with either crude polymyxin P or culture supernatant of M-1 corroborated that polymyxin P is the main component of the biocontrol effect exerted by strain M-1 against phytopathogenic *Erwinia spp*.

## Background

The plant growth-promoting rhizobacterium *Paenibacillus polymyxa*, formerly known as *Bacillus polymyxa*[1], can promote plant growth by producing indole-3-acetic acid (IAA) [2] and volatile compounds [3]. It is also known for controlling plant-parasitic nematodes [4,5] and fungal phytopathogens including *Fusarium oxysporum*[6], *Fusarium graminearum*[7], *Aspergillus niger*[8], *Penicillium expansum*[9], *Leptosphaeria maculans*[10], *Phytophthora palmivora* and *Pythium aphanidermatum*[11]*. P. polymyxa* has been recently used to control bacterial phytopathogens such as *Xanthomonas campestris*[12], and *X. axonopodis*[13]. The antagonistic effect of *P. polymyxa* against phytopathogens is mainly due to its capability to produce antimicrobial substances, such as peptide antibiotics and antimicrobial proteins. *P. polymyxa* can produce several kinds of peptide antibiotics, including polymyxins [14-22], gavaserin and saltavidin [23], jolipeptin [24], gatavalin [25] and fusaricidins [26,27].

Polymyxins which are known for their strong inhibiting effects against gram-negative bacteria have been used to treat multidrug-resistant gram-negative bacteria [28] and to prevent septic shock [29]. The molecular structure of polymyxin is comprised of a cyclic peptide chain and a hydrophobic tail. Each member of polymyxins differs in the structures of fatty acids and the variations in the amino acid residues [30]. Polymyxins are synthesized by the nonribosomal peptide synthetase (NRPS) mechanism [31]. To date, two giant gene clusters responsible for synthesis of polymyxin A [28], and polymyxin B [32] are known.

Among the 202 bacterial strains isolated from surface sterilized wheat plants collected from Beijing and Henan Province, China, one strain designated M-1 was selected due to its inhibiting effect against fungal phytopathogens. Growth of wheat was also enhanced in the presence of this strain indicating its plant growth promoting activity [33]. The whole genome of *P. polymyxa* M-1 has been sequenced, and nine giant gene clusters involved in non-ribosomal synthesis of antimicrobial lipopeptides and polyketides have been detected [34]. Due to its rich spectrum of secondary metabolites with antimicrobial action, *P. polymyxa* M-1 is a good candidate for bio-controlling fire blight, a serious disease in apple and pear caused by *Erwinia amylovora*. Previously, we have shown that the polyketide difficidin and the dipeptide bacilysin produced by *Bacillus amyloliquefaciens* suppress growth of *E. amylovora*[35]. Here, we report that *P. polymyxa* M-1 synthesizes two components of polymyxin P, polymyxin P_1_ and P_2_, which are efficient against *E. amylovora.* Moreover, the corresponding polymyxin synthetase gene cluster in M-1 was identified and further characterized by domain analysis as being different from the *pmx* gene clusters encoding polymyxin A and B, respectively.

## Results

### Characterization of M-1

**Figure 1:**
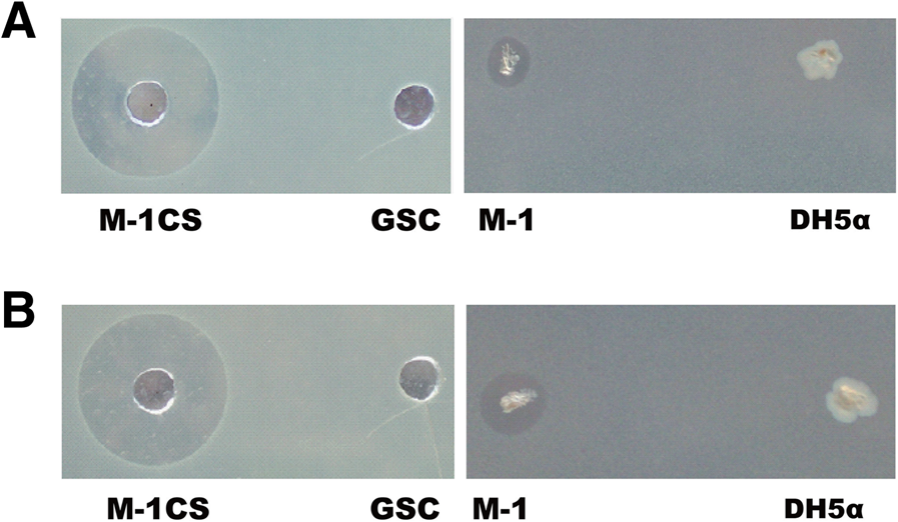
***In vitro *antagonistic effect of *P. polymyxa *M-1 against *E. amylovora *Ea273 and *E. carotovora. *(A)** Inhibiting effect of M-1 culture supernatant (CS) against *E. amylovora* Ea273. **(B)** Inhibiting effect of M-1 culture supernatant against *E. carotovora*. “M-1CS” represents M-1 GSC culture supernatant. GSC medium was used as a negative control. M-1 cells were also spotted onto lawns of *E. amylovora* Ea273 and *E. carotovora*. *E. coli* DH5α cells were used as a negative control.

Culture supernatants of M-1 suppressed growth of several bacteria, including the human opportunistic pathogen *Pseudomonas aeruginosa* (Table 1). Remarkably, growth of phytopathogenic *E. amylovora* Ea 273 and *E. carotovora* was strongly inhibited (Figure 1)*.* M-1 was identified as *P. polymyxa* by its 16S rDNA sequence (gb accession: FR727737) and by physiological and biochemical features. The motile, rod-shaped and spore-forming bacterium was facultative anaerobic, was positive in the Voges-Proskauer reaction (acetylmethylcarbinol), able to hydrolyze starch and to utilize glucose, xylose, glycerol, and mannitol, but did not grow at sodium chloride concentrations exceeding 5%. The whole genome sequence of M-1 (gb accession: HE577054.1) displayed close similarity to the sequences of plant-associated *P. polymyxa* strains SC2 [36] and E681 [3], respectively.

**Table 1:** **Antibacterial activity of *Paenibacillus polymyxa *M-1 culture supernant determined in agar diffusion test**

**Indicator strains**	**Diameter of the inhibition zone (mm)**
*Erwinia amylovora* Ea 273	21.5
*Erwinia carotovora*	20
*Escherichia coli* K12	18
*Pseudomonas aeruginosa*	23
*Streptococcus faecalis*	7
*Micrococcus luteus*	22.5
*Bacillus megaterium*	14.5
*Bacillus subtilis* 168	7.5
*Bacillus amyloliquefaciens* FZB42	6

### Detection and structural characterization of polymyxin P

**Figure 2:**
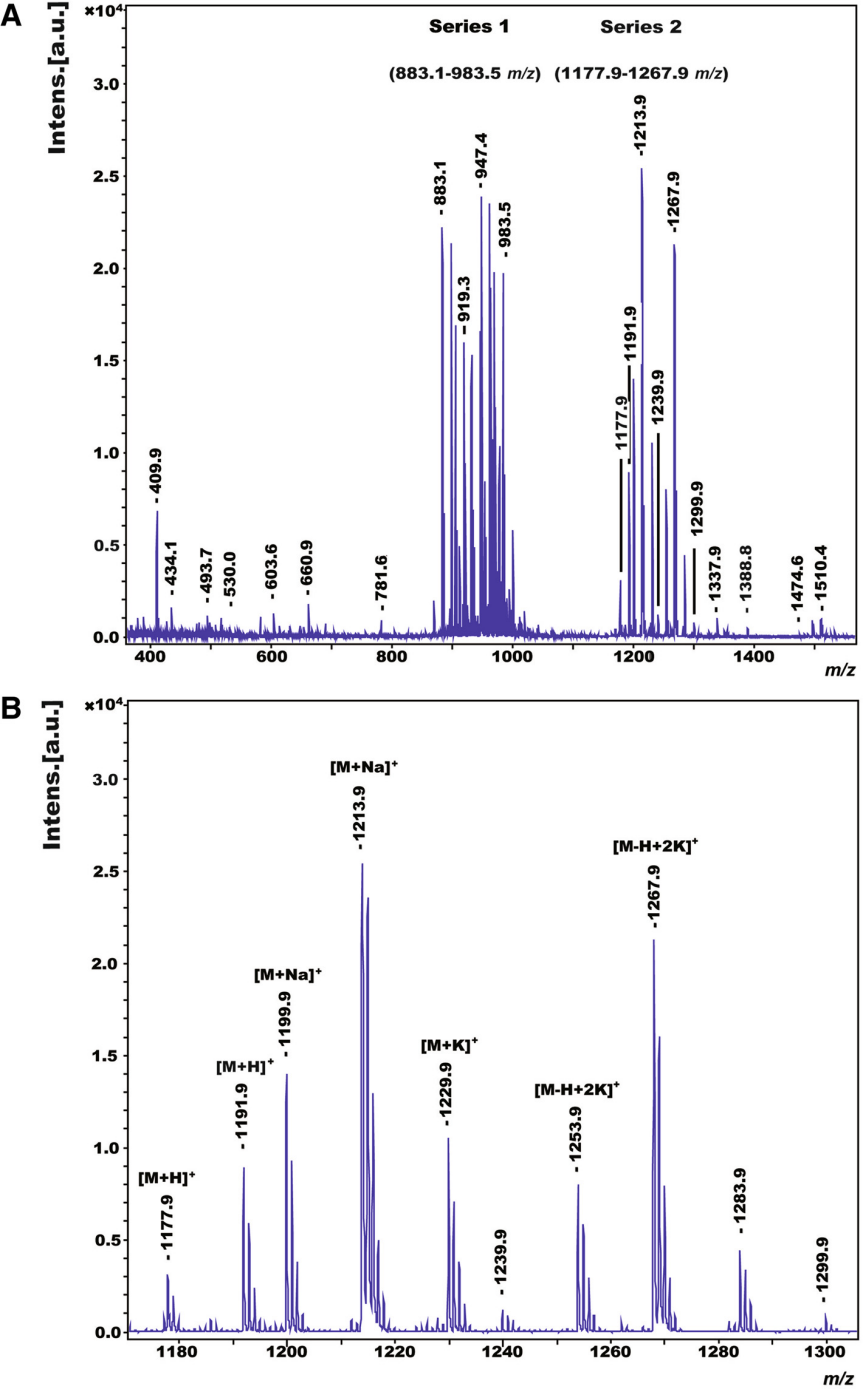
**MALDI-TOF-MS analysis of *P. polymyxa *M-1 secondary metabolites. (A)** Culture supernatant of M-1 grown in GSC medium containing fusaricidin (series 1, from *m/z* = 883.1 to 983.5) and polymyxin P (series 2, from *m/z* = 1177.9 to 1267.9) derived mass peaks. **(B)** Extended view of the mass peaks *m/z* forming series 2. Two polymyxin P metabolites [M + H]^+^*m/z* 1177.9 and 1191.9, and their alkali adducts [M + Na]^+^*m/z* 1199.9 and 1213.9, [M + K]^+^*m/z* 1229.9, and [M-H + 2 K]^+^*m/z* 1253.9 and 1267.9 were distinguished. The nature of the trailing peaks next to the peaks of interest is unknown.

**Figure 3:**
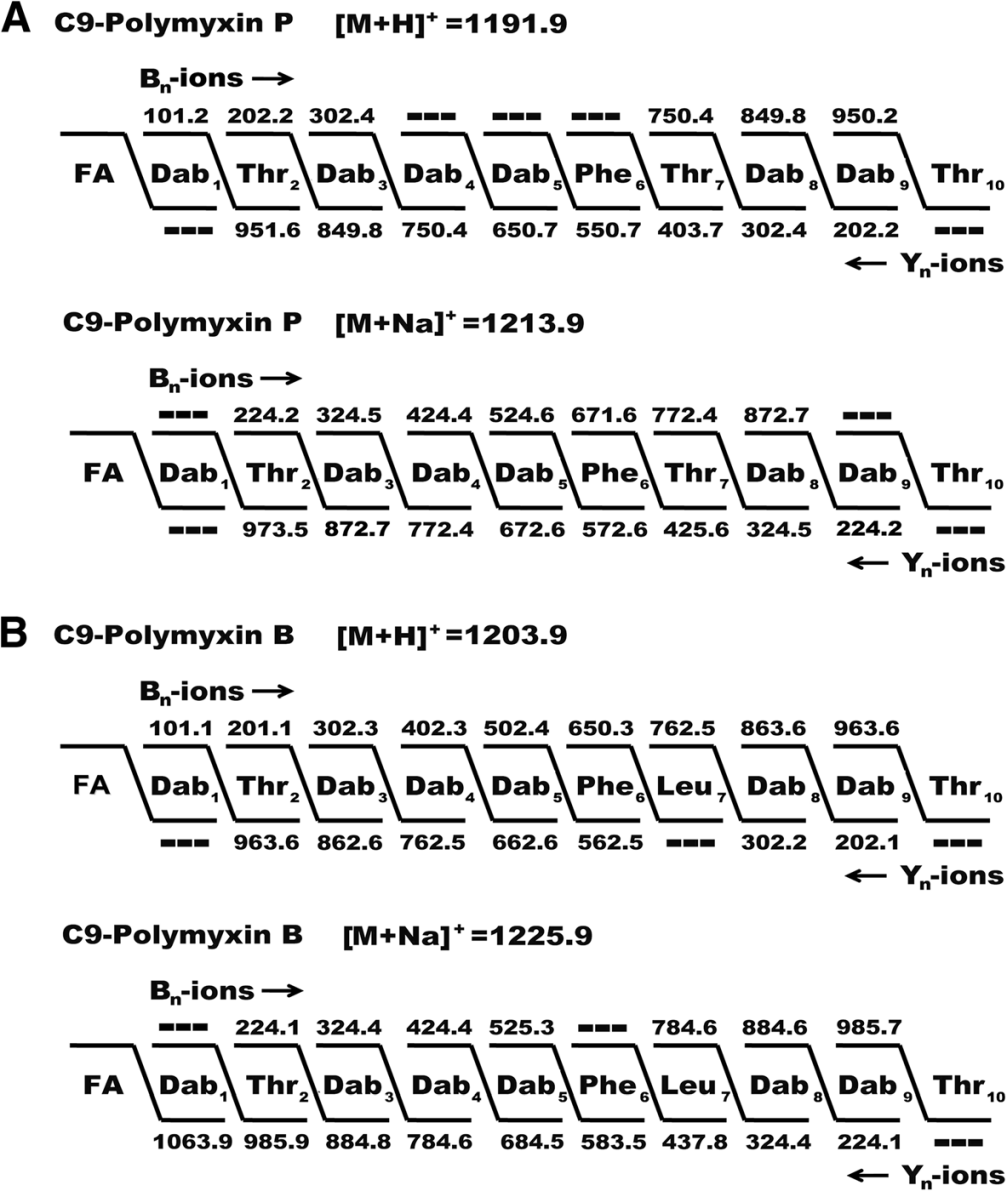
***In situ *structural analysis of polymyxins by PSD-MALDI-TOF mass spectrometry. (A)** Lipopeptide produced by *P. polymyxa* M-1 (with *m/z* of 1191.9 and 1213.9); **(B)** commercial polymyxin B (with *m/z* of 1203.9 and 1225.9) used as the reference. The structures were derived from a series of N- and C-terminal fragments [bn - and Yn -ions]. FA, fatty acid.

The metabolites produced by *P. polymyxa* M-1, possessing antagonistic activities against *E. amylovora* Ea273 and *E. carotovora* were identified by matrix-assisted laser desorption ionization-time of flight mass spectrometry (MALDI-TOF-MS) in combination with bioautography. Antibacterial activities were detected in both cell-surface extracts and a GSC culture supernatant of M-1. Cell surface extracts were prepared by extraction of cells picked from agar plates with 70% acetonitrile/0.1% trifluoroacetic acid [37]. By MALDI-TOF-MS, two prominent series of mass peaks were detected, ranging from *m/z* = 883.1 to 983.5 (series 1) and from *m/z* = 1177.9 to 1267.9 (series 2) (Figure 2A), respectively. Members of series 1 were attributed to the well-known fusaricidins (unpublished data), a family of lipodepsipeptides exhibiting potent antifungal activities [38]. The compounds of series 2 (Figure 2B) were investigated by MALDI-TOF-MS in more detail. Two metabolites were detected, of which the protonated forms showed masses of *m/z* = 1191.9 and *m/z* = 1177.9. The other mass peaks of series 2 could be attributed to the alkali adducts of these compounds as indicated in Figure 2B. Their structures were determined by postsource decay (PSD)-MALDI-TOF-MS analysis and compared with the fragment spectrum of polymyxin B which was commercially available (Figure 3).

The fragment spectra of both the M-1 products of series 2 and polymyxin B as the reference revealed the presence of imino ions of threonine (*m/z* = 74.1) and phenylalanine (*m/z* = 120.3) as well as dipeptide ions of Dab-Dab (2,4 diaminobutyric acid, *m/z* = 201.4), Dab-Thr (*m/z* = 202.2) and Dab-Phe (*m/z* = 248.3). The M-1-products and polymyxin B differed in the dipeptide fragments Phe-Thr (*m/z* = 249.4) (M-1) and Phe-Leu (*m/z* = 261.1) (polymyxin B). These comparative nearest neighbour relationships imply that the compounds of series 2 belong to the polymyxin family which are well known antibiotics produced by *P. polymyxa*. This conclusion was confirmed by fragment analysis using PSD-MALDI-TOF mass spectrometry. Figure 3 shows the peptide sequence of the M-1 metabolite with the mass number of *m/z* = 1191.9 and the polymyxin B with *m/z* = 1203.9 as well as of their sodium adducts. In each case the best results were accomplished in mass spectrometric sequencing, when a break of the peptide bond between residue 4 and the C-terminus is assumed. The sequence of the resulting linearized peptide follows residues 1–10. The most significant and almost complete sequence information was obtained in the case of the b_n_ – ions, when fragmentation starts between Dab_1_ and Thr_2_. For the Y_n_ – ions the best results were achieved, when fragmentation begins between Thr_10_ and Dab_9_. In this way -Dab_1_-Thr_2_-Dab_3_-Dab_4_-Dab_5_-Phe_6_-Thr_7_-Dab_8_-Dab_9_-Thr_10_- was determined as the peptide sequence of the two M-1 – metabolites of series 2, which can be attributed to polymyxin P containing Phe, Thr and Dab in a molecular ratio of 1 : 3 : 6 [14]. In this way, these metabolites could be identified as two isomers of polymyxin P, designated as polymyxin P_1_ and P_2_. The mass spectrum of the reference compound polymyxin B also showed two mass peaks at *m/z* = 1189.3 (B1) and 1203.9 (B2). They were attributed to two variants of polymyxin B differing in their fatty acid component, which is either an iso-octanoyl (C_8_H_15_O) or a 6-methyloctanoyl (anteisononanoyl, C_9_H_17_O) residue [21,32]. By comparison with polymyxin B and other members of the polymyxin family, we conclude that polymyxin P_1_ and P_2_ from strain M-1 contain the same fatty acid residues consistent with the data reported by Kimura et al. for polymyxin P [14].

### The anti-*Erwinia* activity of polymyxin P produced by *P. polymyxa* M-1

**Figure 4:**
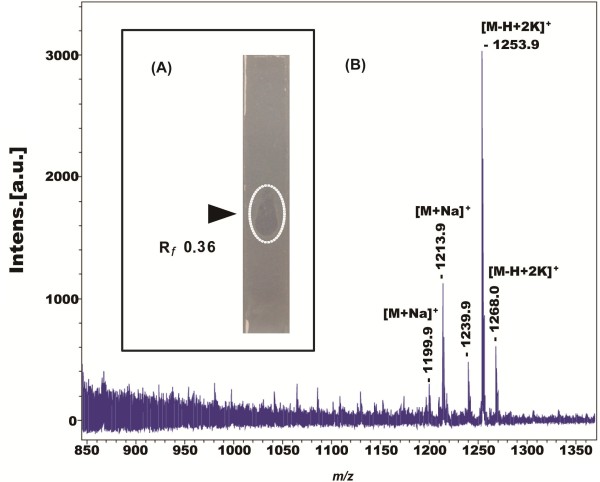
**Detection of the anti-*Erwinia *metabolite produced by *P. polymyxa *M-1. (A)** Detection of the antibacterially acting metabolite by bioautography. Supernatants prepared from strain M-1 grown in GSC medium for 36 h were separated by TLC and sandwiched with indicator strain *E. carotovora.* The inhibiting band at R_*f*_ 0.36 was circled. **(B)** MALDI-TOF-MS analysis of the antibacterial compounds detected by bioautography.

In order to identify the compounds which suppress the growth of *E. amylovora* Ea273 *and E. carotovora* in M-1 GSC culture, the supernatant was subjected to thin layer chromatography (TLC) in combination with bioautography [39] (Figure 4). One spot exhibiting antibacterial activity was observed at R_*f*_ 0.36 (Figure 4A) which was identical with that of polymyxin P [14]. It was scraped off from the thin layer plate. The silica gel powder obtained was extracted with methanol, and the extract was analyzed by MALDI-TOF-MS. The obtained mass spectrum ranging from *m/z* = 850 to 1350 (Figure 4B) indicates the same mass peaks at *m/z* = 1199.9, *m/z* = 1213.9, *m/z* = 1239.9, *m/z* = 1253.9 and *m/z* = 1268.0 as previously been detected for series 2 in Figure 2. From these results we conclude, that polymyxin P_1_ and P_2_ represent the active compounds inhibiting growth of the *Erwinia* test strains. There were no mass signals pointing to fusaricidines (*m/z* = 850 – 1000) or other metabolites showing antibacterial activity (Figure 4B). Thus, polymyxin P was proven to be an anti-*Erwinia* metabolite which was produced by M-1.

**Figure 5:**
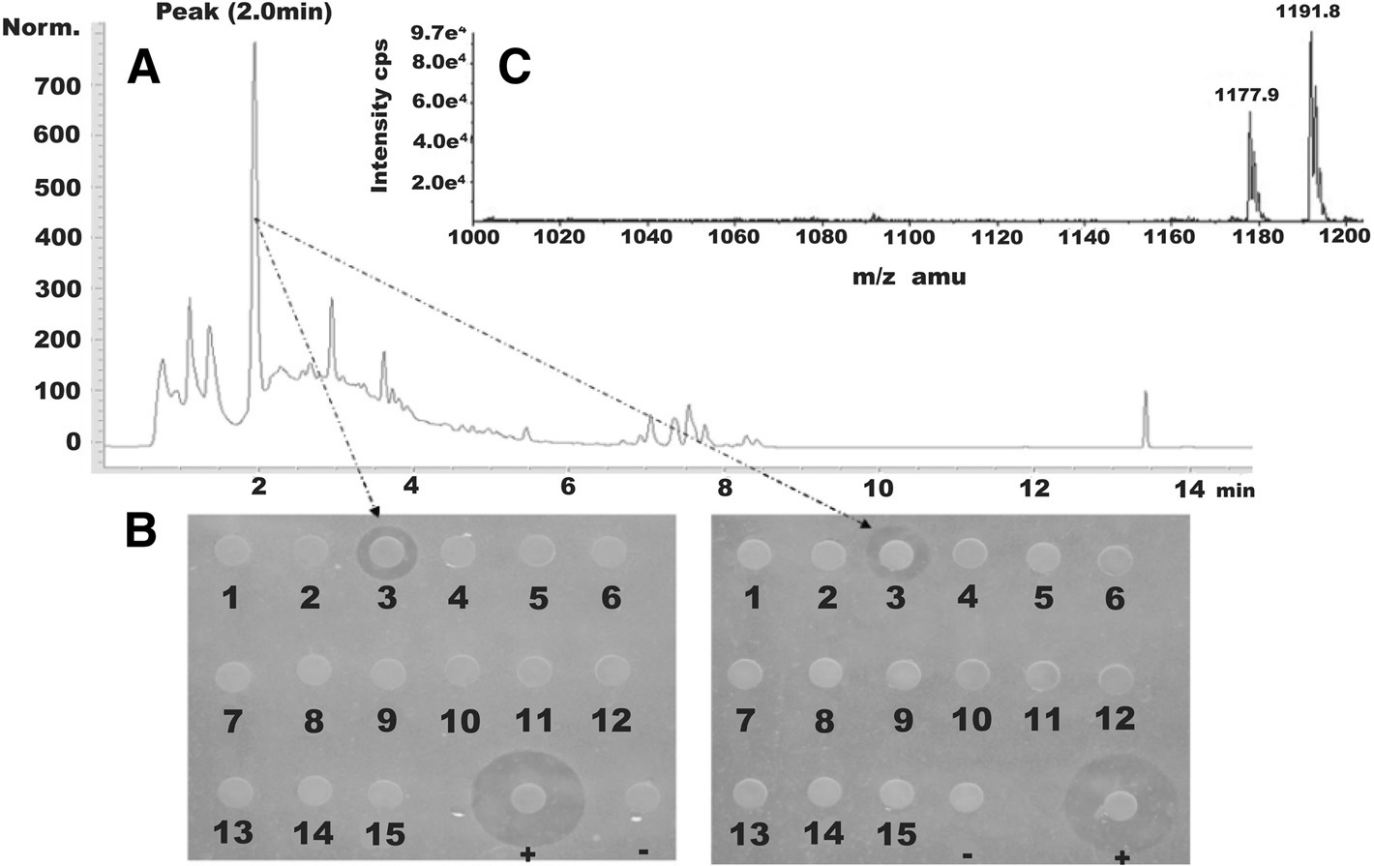
**RP-HPLC analysis and antibacterial activity test of fractions. (A)** RP-HPLC (HPLC type: Agilent 1100) analysis of M-1 GSC culture supernatant using a Luna C_18_ column (100 Å 150 × 4.6 mm, Phenomenex, Aschaffenburg, Germany). **(B)** Antibacterial activity test of HPLC fractions of M-1 GSC culture supernatant. 15 paper discs loaded with HPLC fractions were put onto LB agar mixed with the two *Erwinia* strains. The numbers below paper discs indicate different fractions. Fractions 3 corresponding to the peak at retention time 2 min in the M-1 culture supernatant HPLC chromatogram showed antagonistic effects against the growth of *E. amylovora* Ea273 (left) and *E. carotovora* (right). “ + ” represents positive control, discs loaded with M-1 culture supernatant, while “-” represents negative control, discs loaded with sterile water. **(C)** HPLC-ESI-MS analysis of fraction 3.

To corroborate these results, a GSC culture supernatant of M-1 was fractionated by reversed-phase high-performance liquid chromatography (RP-HPLC) (Figure 5A). Fifteen fractions were obtained. The fraction appearing at a retention time of 2 displayed antagonistic effects against the growth of the two phytopathogenic *Erwinia* indicator strains (Figure 5B). This fraction was analyzed by high-performance liquid chromatography electrospray ionization mass spectrometry (HPLC-ESI-MS). Two peaks were detected at *m/z* = 1191.8 and *m/z* = 1177.9, which also correspond to the two isomers of polymyxin P [14] (Figure 5C).

### Morphological changes of *Erwinia* strains caused by treatment with crude polymyxin P

**Figure 6:**
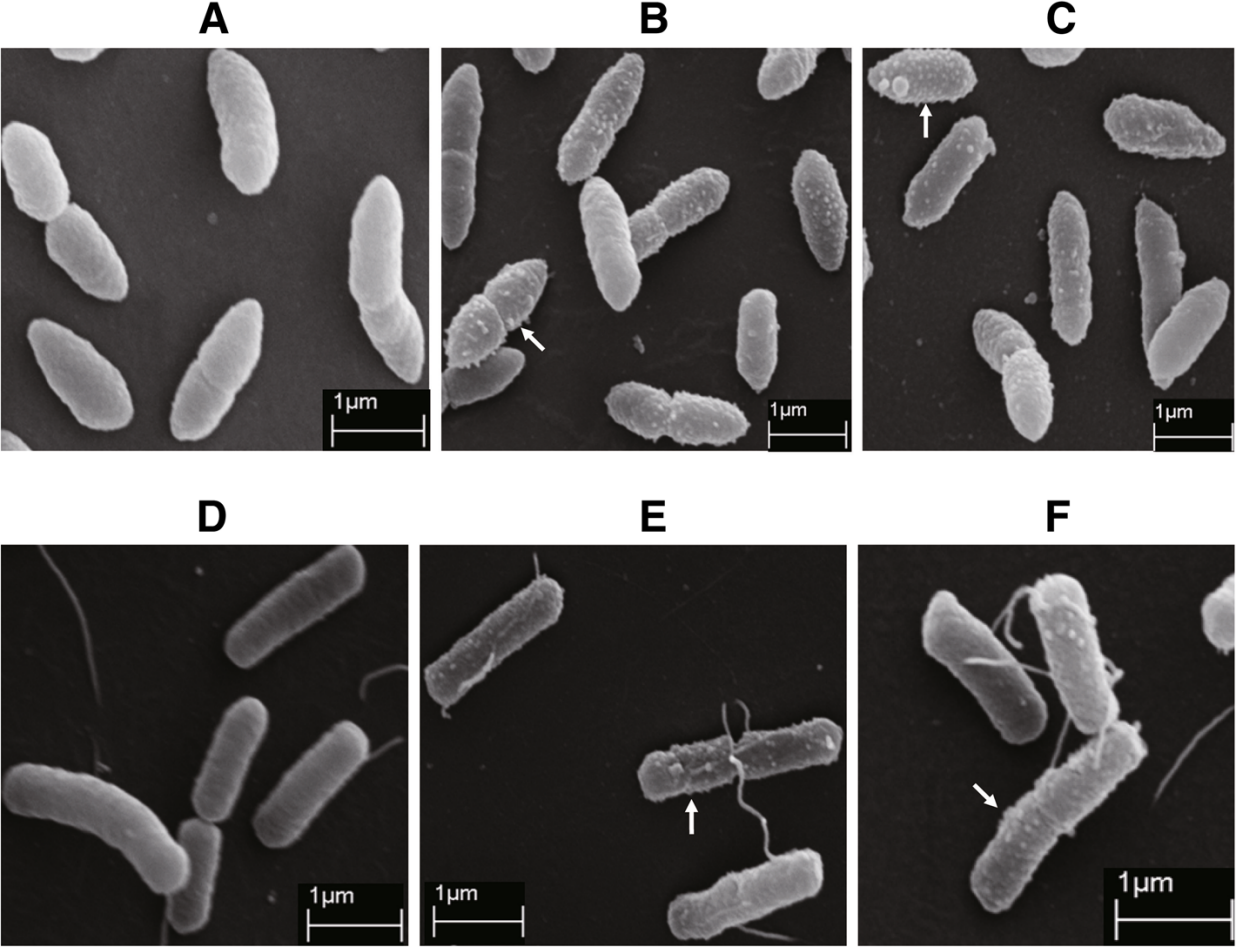
**Morphological changes of the *Erwinia *strains treated with polymyxin P and M-1 GSC culture supernatant. (A)** Untreated *E. amylovora* Ea273; **(B)***E. amylovora* Ea273 treated with crude polymyxin P; **(C)***E. amylovora* Ea273 treated with M-1 GSC culture supernatant; **(D)** Untreated *E. carotovora*; **(E)***E. carotovora* treated with crude polymyxin P; **(F)***E. carotovora* treated with M-1 GSC culture supernatant. Protrusions on cell surfaces of *E. amylovora* Ea273 and *E. carotovora* treated with crude polymyxin P and M-1 GSC culture supernatant were marked by arrows.

The effect of the crude polymyxin P prepared by RP-HPLC described above against two phytopathogenic *Erwinia* strains was studied by scanning electron microscopy (SEM). Cell surfaces of both untreated *E. amylovora* Ea 273 and *E. carotovora* appeared smooth without any visible irregularities (Figure 6A and D). However, dense projections were observed on cell surfaces of the two phytopathogens treated with crude polymyxin P (Figure 6B and E) or cell- free supernatant prepared from M-1 GSC culture (Figure 6C and F) suggesting that polymyxin P caused the same morphological change as M-1 GSC culture supernatant did. Similar morphological changes were also found on cell surfaces of *Salmonella typhimurium, Escherichia coli* B [40], *Chlamydia psittaci*[41] and *Pseudomonas aeruginosa* treated with polymyxin B or E [42].

The observed morphological changes at the surface of the *Erwinia* cells treated with polymyxin support an action mechanism in which polymyxin, bound at the lipopolysaccharide component of the outer membrane (OM), does permeabilize the OM [30] and - as shown here - generates visible protrusions.

### Characterization of the gene cluster encoding polymyxin biosynthesis in *P. polymyxa* M-1

**Figure 7:**
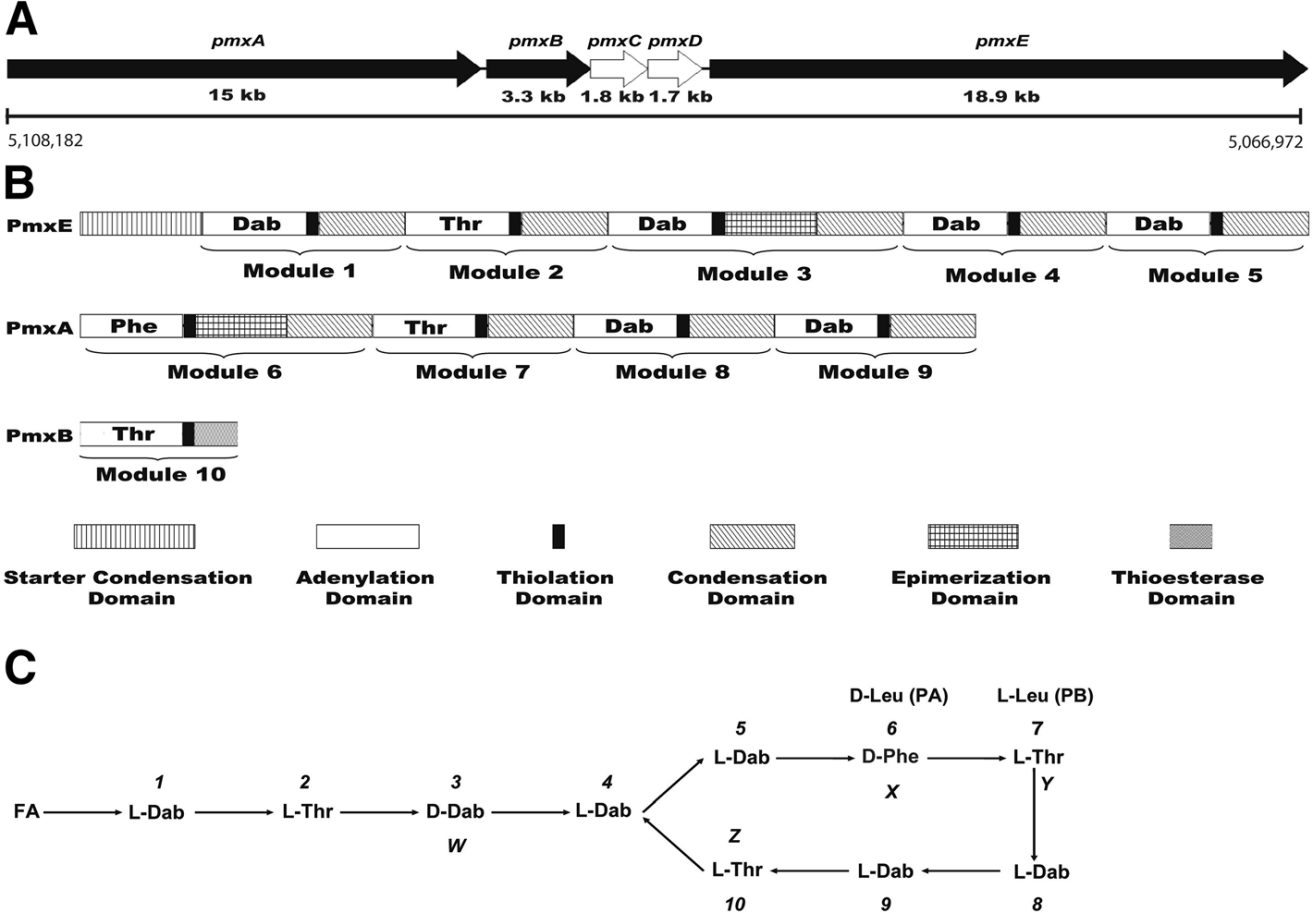
**Putative gene cluster for polymyxin biosynthesis in *P. polymyxa *M-1 and primary structure of polymyxin P. (A)** Genetic structure of the *pmx* genes. Black filled arrows represent NRPS genes, while white arrows represent ABC transporter-like genes. The position of the gene cluster within the chromosome of M-1 is indicated. **(B)** Domain organization of the putative Pmx enzymes. **(C)** Primary structure of polymyxin P synthesized in *P. polymyxa* M-1 derived by bioinformatic and chemical analysis. FA, fatty acid, 6-methyloctanoic acid or isooctanoic acid. “1-10” indicate the ten amino acid moieties. Four variable sites were marked as “W, X, Y and Z”, respectively. Phe at the sixth position (X) of polymyxin P is replaced by Leu at the corresponding position of polymyxin A [28], while Thr at the seventh position (Y) of polymyxin P is substituted by Leu at the corresponding position of polymyxin B [32]. Polymyxin A and polymyxin B are labelled as “PA” and “PB”, respectively.

The genome of *P. polymyxa* M-1 contains a 41 kb gene cluster displaying overall identities of 96.41% to the well characterized polymyxin synthetase gene cluster from *P. polymyxa* E681 [28] and of 91.2% to that from *P. polymyxa* PKB1 [32] on the nucleotide sequence level. Corresponding to the *pmx* gene clusters of E681 and PKB1, the M-1 gene cluster consisted of five open reading frames, *pmxA*, *pmxB*, *pmxC*, *pmxD* and *pmxE* (Figure 7A). Three of them, the genes *pmxA*, *pmxB* and *pmxE* were directly involved in non-ribosomal peptide synthesis, while *pmxC* and *pmxD* were encoding ABC transporters (ATPase and permease components).

Domain analysis performed with the NRPSpredictor2 server of the university of Tuebingen [43] revealed that the putative polymyxin synthetase of M-1 comprises ten modules (Figure 7B). Each of them consists of three or four domains, such as A-T-C, A-T-E-C or A-T-TE. However, similar to the *pmx* gene clusters in *P. polymyxa* PKB1 and *P. polymyxa* E681, the order and arrangement of the NRPS encoding genes was not collinear with the amino acids in the polymyxin end product. PmxA, a polypeptide containing 5010 amino acids, comprised four modules. The substrate specificities of the four adenylation domains (A-domain) were predicted to activate the amino acid substrates D-Phe-6, L-Thr-7, L-Dab-8 and L-Dab-9, respectively. PmxB, a polypeptide consisting of 1102 amino acids, contained the remaining part of the last module including a thioesterase domain (TE-domain), A-T-TE. The A-domain was predicted to activate L-Thr-10. PmxE, a 6312 amino-acid polypeptide, contained five modules responsible for the first five amino acids of polymyxin P. In addition, a N-terminal condensation domain with similarity to starter C-domain simultaneously acylating the first amino acid with a fatty acid tail was identified [44]. The five A-domains were predicted to activate L-Dab-1, L-Thr-2, D-Dab-3, L-Dab-4, and L-Dab-5, respectively. Therefore, the ten modules were arranged in the gene order *pmxE*-*pmxA*-*pmxB* (Figure 7B). There were two epimerization domains (E-domains), occurring in the third and sixth module, which indicated that the third and sixth amino acid of the polymyxin produced by M-1 represented D-forms, D-Dab and D-Phe, respectively. The TE-domain located at the carboxy-terminal region of PmxB was probably responsible for terminating polymyxin synthesis by cyclization and releasing the product. The domain organization analysis of the putative polymyxin synthetase from M-1 implied that the lipopeptide synthesized by the synthetase is identical with polymyxin P (Figure 7C) [14], which coincides with the results obtained by mass spectrometric analysis. Although there is high overall sequence similarity between the polymyxin gene clusters of M-1, E681, and PKB1, the A domains in modules 6(X) and 7(Y) activate different amino acids. The identity between the amino acid sequences of the sixth modules of polymyxin synthetases of M-1 and E681, activating Phe and Leu, respectively, was only 88%. An even lower identity of 51% on the amino acid level was found for the A-domains of the seventh module in the polymyxin synthetases from M-1 and PKB1, activating either Thr or Leu, respectively.

Polymyxin antibiotics are lipopeptides, and as in case of the two other known *pmx* gene clusters, no genes were found in the vicinity of the *pmx* gene cluster of *P. polymyxa* M-1 which might be involved in lipidation of the peptide moiety. It is likely that polymyxin synthesis resembles surfactin synthesis, and relies upon lipidation functions encoded elsewhere in the chromosome [32]. Notably, a thioesterase-like gene, *pteH* (COG3208), bearing a GrsT domain and similar to *Bacillus amyloliquefaciens* SrfAD (27% identity), was preceding a giant peptide synthetase gene at 2,508,313 in the genome of M-1. However, the PteH protein contains no acyltransferase domain and its role in attaching the fatty acid moiety to the polymyxin dekapeptide remains to be elusive.

## Discussion

In this study, we found that growth of two important phytopathogens, *E. amylovora* Ea273 and *E. carotovora* was inhibited by M-1. Polymyxin P was identified as being the active principle of M-1. Two lines of evidence supported this finding: (1) M-1 supernatants formed a distinct clearing spot when exposed to bioautography using the *Erwinia* strains as indicator. When the material isolated from that area was analyzed by MALDI-TOF mass spectroscopy, the mass peaks with *m/z* of 1199.9, 1213.9, 1253.9 and 1268.0 indicating alkali adducts of polymyxin P were detected (Figure 4); (2) a single fraction obtained by HPLC contained the inhibiting activity against bacterial pathogens and also the characteristic mass peaks *m/z* were indicating the presence of polymyxin P in this sample (Figure 5).

Polymyxin P is a peptide antibiotic reported more than 40 years ago, and two species with different hydroxy fatty acids were described. Polymyxin P_1_ contains anteisononanoic acid, a-C_9_, and polymyxin P_2_ isooctanoic acid, i-C_8_[14]. Although its constituent amino acids have been determined as being six Dab, three Thr, and one Phe; to the best of our knowledge, no further investigation about the primary structure of polymyxin P and the configuration of the constituent amino acids has been performed until now.

Here we established the primary structure of polymyxin P by PSD-MALDI-TOF mass spectrometry (Figure 3). Alterations in comparison to other polymyxin species were detected in two out of the four variable positions of the peptide. A unique Phe residue resided at the sixth position (X), and a Thr residue was found at the seventh position (Y) of polymyxin P. These results were corroborated by bioinformatic analysis of the polymyxin synthetase gene cluster in M-1, where the adenylation domains specified the amino acid substrates to be activated (Table 2). The resulting order of amino acids did completely match with the structure for polymyxin P obtained by PSD MALDI-TOF MS (Figure 7C). Whilst this technique did not deliver information about stereochemical configuration of the amino acid moieties, our bioinformatic approach resulted in detection of two epimerization-domains residing in the third and the sixth module (Figure 7B), suggesting the presence of D-Dab and D-Phe in position 3 and 6 of the polymyxin product, respectively (Figure 7C). The occurrence of D-Dab in position 3 corresponds with recent findings in polymyxin A [28] and polymyxin B [32]. This is remarkable, since according to literature, these forms of polymyxin are rare and the fact that all three of the polymyxin gene clusters examined to date are from plant-associated strains of *P. polymyxa* isolated for their biocontrol and plant growth promoting activities is relevant for this observation [32].

**Table 2:** Specificity-conferring amino acids and homologies of the adenylation domains in polymyxin synthetases of strains M-1, E681, and PKB1

**Module/ strain**	**Active site residues in A-domain**								**Specified aa**	**% aa E681**	**% aa PKB1**
	**235**	**236**	**239**	**278**	**299**	**301**	**322**	**330**			
*Module 1*											
**pmxE1/M-1**	D	V	G	E	I	S	S	I	**L-Dab**	99	99
pmxE1/E681	D	V	G	E	I	S	S	I	L-Dab		
pmxE1/PKB1	D	V	W	E	I	S	S	I	L-Dab		
*Module 2*											
**pmxE2/M-1**	D	F	W	N	I	G	M	V	**L-Thr**	99	98
pmxE2/E681	D	F	W	N	I	G	M	V	L-Thr		
pmxE2/PKB1	D	F	W	N	I	G	M	V	L-Thr		
*Module 3 (W)*											
**pmxE3/M-1**	D	V	G	E	I	S	S	I	**D-Dab**	98	92
pmxE3/E681	D	V	G	E	I	S	S	I	D-Dab		
pmxE3/PKB1	D	V	G	E	I	S	S	I	D-Dab		
*Module 4*											
**pmxE4/M-1**	D	V	G	E	I	S	A	I	**L-Dab**	96	96
pmxE4/E681	D	V	G	E	I	S	A	I	L-Dab		
pmxE4/PKB1	D	V	G	E	I	S	A	I	L-Dab		
*Module 5*											
**pmxE1/M-1**	D	V	G	E	I	S	A	I	**L-Dab**	97	89
pmxE1/E681	D	V	G	E	I	S	A	I	L-Dab		
pmxE1/PKB1	D	V	G	E	I	S	A	I	L-Dab		
*Module 6 (X)*											
**pmxA1/M-1**	D	A	W	T	I	A	A	I	**D-Phe**	88	99
pmxA1/E681	D	A	W	I	V	G	A	I	D-Leu		
pmxA1/PKB1	D	A	W	T	I	A	A	I	D-Phe		
*Module 7 (Y)*											
**pmxA2/M-1**	D	F	W	N	I	G	M	V	**L-Thr**	99	51
pmxA2/E681	D	F	W	N	I	G	M	V	L-Thr		
pmxA2/PKB1	D	G	F	L	L	G	L	V	L-Leu		
*Module 8*											
**pmxA3/M-1**	D	V	G	E	I	S	A	I	**L-Dab**	97	92
pmxA3/E681	D	V	G	E	I	S	A	I	L-Dab		
pmxA3/PKB1	D	V	G	E	I	S	A	I	L-Dab		
*Module 9*											
**pmxA4/M-1**	D	V	G	E	I	S	A	I	**L-Dab**	96	91
pmxA4/E681	D	V	G	E	I	S	A	I	L-Dab		
pmxA4/PKB1	D	V	G	E	I	S	A	I	L-Dab		
*Module 10 (Z)*											
**pmxB1/M-1**	D	F	W	N	I	G	M	V	**L-Thr**	97	99
pmxB1/E681	D	F	W	N	I	G	M	V	L-Thr		
pmxB1/PKB1	D	F	W	N	I	G	M	V	L-Thr		

Modules 3 and 6 contained extra epimerization domains which might convert Dab3 and Phe6 to the D-configuration. A-domains in modules 6 (X) and 7(Y) specifying different amino acids in the polymyxins produced by M-1, E681, and PKB1 are underlined.

## Conclusions

Our results support the view that polymyxin P encoded by the *pmx*ABCDE gene cluster is the main compound in the culture filtrate of *P. polymyxa* M-1 in suppressing *E. amylovora* and *E. carotovora*, the causative agents of the important plant diseases fire blight and soft rot, respectively. Since the rare polymyxin P has not been previously used as a clinical agent, in contrast to polymyxin B and colistin [30], this finding provides a potential option to use polymyxin P or its producer strain *P. polymyxa* M-1 as an alternative of chemical bactericides to control fire blight, soft rot and other plant diseases caused by gram-negative bacteria.

## Methods

### Bacterial strains and growth conditions

Strain M-1 isolated from surface sterilized wheat roots in China was kept frozen at −70 C with 15% glycerol as a laboratory stock. This strain was cultured in tryptic soy broth (TSB) liquid medium or on tryptic soy broth agar (TSBA) plates (TSB supplemented by 1.5% agar) at 30°C for general purposes or in glucose-starch-CaCO_3_ (GSC) medium [45] at 30°C for antibacterial activity tests and chemical analysis of polymyxin. M-1 has been deposited in China General Microbiological Culture Collection Center (CGMCC) as strain CGMCC 7581. Other strains used in this study were laboratory stocks obtained from different sources and kept frozen with 15% (v/v) glycerol at −70°C. They were grown in Luria broth (LB) or on LB agar plates (LB solidified with 1.5% agar) at 30°C (*E. amylovora* Ea273, *E. carotovora* and *Micrococcus luteus*) or 37°C (*Pseudomonas aeruginosa, Streptococcus faecalis, Bacillus megaterium, Bacillus subtilis* 168, *Bacillus amyloliquefaciens* FZB42 and *Bacillus cereus* ATCC 14579).

### Bacterial identification

Identification of the strain M-1 was carried out by using 16S rDNA sequence analysis as well as by physiological and biochemical characterization. After growing in TSB medium at 30°C overnight, the bacteria cells were collected by centrifuging for chromosomal DNA isolation using the standard phenol:chloroform procedure. Then, the 16S rDNA was amplified by PCR with two pairs of primers 63 F (5’CAG GCC TAA CAC ATG CAA GTC-3’), 1387R (5’GGG CGG TGA TGT ACA AGG C’-3) [46], 530 F (5’GTG CCA GCM GCC GCG G-3’) and 1494R (5’GGY TAC CTT GTT ACG ACT T-3’) [46,47]. The reaction mixture included Taq DNA polymerase, 10 × Taq buffer, forward and reverse primers, each deoxynucleoside triphosphate (dATP, dGTP, dCTP and dTTP) (Beijing Youbo Gene Technology Co., Ltd) and template DNA. Amplifications were performed using a Biometra T personal 48 thermocycler (Biometra, Goettingen, Germany) with the following cycle conditions: initial activation at 94°C for 5 min; 35 cycles of 94°C for 1 min, 55°C for 30 sec, and 72°C for 1 min; a final extension at 72°C for 10 min. PCR products (100 μL total volume) were analyzed by electrophoresis using a 0.8% (w/v) Tris-acetate-EDTA (TAE) agarose gel mixed with ethidium bromide and ultraviolet visualization. PCR products were purified from ethidium bromide-stained gels using the DNA product purification Kit (TIANGEN BIOTECH (BEIJING) CO., LTD) and inserted into pMD18-T vectors (TAKARA BIOTECHNOLOGY (DALIAN) CO., LTD). The recombinant plasmids were transformed into *E.coli* DH5α. DNA sequencing of the plasmids was done by Beijing Youbo Gene Technology Co. Ltd. Nucleotide sequences were assembled and edited with Gap4 which is a part of the STADEN package (http://staden.sourceforge.net/) software. The sequences were compared with those of the reference organisms by Blast search.

### Selection of a medium for polymyxin production

Among the seven media used in our survey, Katznelson and Lochhead (KL) medium [48], Landy medium [49], Landy medium either supplemented with yeast extract, D, L-alanine and phenylalanine (Landy GA) or with yeast extract and phenylalanine (Landy G), GSC medium [45], brain-heart infusion (BHI) medium and tryptic soy broth yeast extract (TSBYE) medium, the GSC medium was optimal for production of polymyxin.

### Antibacterial activity assay

To investigate its antibacterial spectrum, *P. polymyxa* M-1 was grown in GSC medium under aerobic conditions at 30°C for 72 h. Then the culture was centrifuged at 6000 rpm at 4°C for 10 min to remove cells. Fresh indicator bacteria plates were prepared for the assay. When the concentration of indicator bacteria grown in LB medium at appropriate temperature was up to 4 × 10^7^ CFU/mL, 0.5 mL bacteria suspension was mixed with 20 mL melting LB agar and cooled below 60°C to prepare the plates. 50 μL M-1 GSC culture supernatant were loaded into a well punched in indicator bacteria plate which was then incubated at 30°C overnight to observe the growth inhibition effect. GSC medium without bacteria was also loaded as a negative control. The diameters of inhibition zones were then measured and recorded.

The inhibiting activity of M-1 against *E. amylovora* Ea273 and *E. carotovora* was also tested by spotting bacterium on an indicator bacteria plate prepared by the method described above. *E. coli* DH5α used as a negative control was also spotted onto the lawn of indicator strains. Then the plates were incubated at 30°C overnight to observe the growth inhibition effect.

To analyze the antibacterial activity of the HPLC fractions, a 50-μL aliquot of each fraction was loaded onto sterilized paper disks. 50 μL M-1 GSC culture supernatant used as a positive control and 50 μL sterile distilled water used as a negative control were also loaded. After being air dried in a clean bench, the disks were transferred onto *E. amylovora* Ea273 and *E. carotovora* plates prepared by the method described above and incubated at 30°C overnight to observe growth inhibition effect.

### Separation of antibacterial compounds by RP-HPLC

The chromatographic system consisted of an Agilent 1100 liquid chromatograph equipped with a diode-array detector (Agilent Technologies, Waldbronn, Germany). Hundred μL M-1 culture supernatant were applied to the RP-HPLC column (Luna C18 100 Å 150 mm × 4.6 mm, Phenomenex, Aschaffenburg, Germany) and eluted isocratically with H_2_O containing 0.1% HCOOH at a flow rate of 1 mL/min. The obtained fractions were freeze-dried, dissolved in sterile distilled water and subjected to an antibacterial test described above. The active fraction was subsequently used for high performance liquid chromatography electrospray ionization mass spectrometry (HPLC-ESI-MS) analysis.

### Bioautography

Bioautography was performed as previously described [39]. In short, M-1 GSC culture supernatant was loaded onto an XAD16 (Sigma) resin column which was then washed and eluted with methanol. After being dried by a rotary evaporator, the samples were redissolved in methanol and spotted onto silica gel 60 F254 thin-layer chromatography (TLC) aluminium sheets (20 by 20 cm; Merck, Darmstadt, Germany) and separated by TLC using *n*-BuOH : AcOH : H_2_O = 4:1:3 containing 1/20 volume of pyridine as the solvent system. Afterwards, strips of the TLC plates were stuck on the surface of the LB agar containing indicator strains at room temperature for 2 h. The LB agar plates were then incubated at 30°C overnight. The inhibition zones documented the positions of the antibacterial compounds separated by TLC. Their R_*f*_ values were calculated. The experiments were repeated at least three times. Matrix material from the positions at which the antibacterial compounds were located was scraped from the silica gel, and extracted with methanol. Then the extracts were lyophilized and analyzed by MALDI-TOF-MS.

### MS analysis

Metabolites in culture supernatant of M-1 were investigated by MALDI-TOF-MS. After M-1 was grown in GSC medium at 30°C for 72 h, samples for mass spectrometric analysis were taken from the culture supernatant and used for measurements after dilution 1:10 with 50% acetonitrile: 50% water containing 0.1% trifluoroacetic acid (solution A). Samples from the TLC plates were diluted in the same way.

MALDI-TOF-mass spectra were recorded using a Bruker Autoflex instrument equipped with a 337 nm nitrogen laser for desorption and ionization. A 2-μL aliquot of each sample was mixed with the same volume of matrix solution (a saturated solution of α-cyano-4-hydroxycinnamic acid in solution A), spotted on the target, air dried and measured as described previously [50].

Spectra were recorded by positive ion detection in reflector mode. The acceleration and reflector voltages were 19 and 20 kV in pulsed ion extraction mode. A molecular gate of 350 Da improved the measurements by filtering out most matrix ions. PSD-MALDI-TOF-MS of the polymyxins were generated with the same samples. Monoisotopic masses were obtained.

In addition, M-1 GSC culture supernatant and the active fraction were analyzed by an online HPLC (1100 series HPLC system, Agilent Technologies) coupled to a QTRAP 2000 mass spectrometer (Applied Biosystems) using a Luna C_18_ 100 Å 50 × 1 mm column (Phenomenex). Samples were applied to HPLC-ESI-MS by isocratic elution with H_2_O containing 0.1% formic acid at a flow rate of 60 μL/min in 10 min. MS analysis was performed in positive ion mode with a mass window ranging from *m/z* 500–1400.

### Polymyxin treatment

The *Erwinia* strains were treated with crude polymyxin P by the method described previously [51] with some modification. The crude polymyxin P (final concentration: 20 μg/mL) or GSC culture supernatant of M-1 (final concentration: 1% (v/v)) was added to LB cultures of the *Erwinia* strains at OD_600nm_ of 0.1. After being inoculated at 28°C for 2 h, the suspensions were centrifuged at 4000 rpm for 5 min to collect bacteria which were then washed two times before observation by SEM.

### Scanning electron microscopy

For analysis by SEM, cells were spinoculated on poly-lysine coated cover glasses and fixed with 2.5% glutaraldehyde/2% para-formaldehyde in 100 mM cacodylate buffer (pH 7.4) at 4°C overnight. After fixation cells were rinsed three times for 10 minutes with 100 mM cacodylate buffer, postfixed for 3 h in 1% osmiumtetroxide, rinsed again three times for 10 minutes with 100 mM cacodylate buffer and dehydrated through an ethanol series. After critical point drying, cells were coated with gold and analyzed on an LEO 1430 scanning electron microscope.

## Competing interests

The authors declare that they have no competing interests.

## Authors’ contributions

BN carried out the main experiments, data analysis and wrote a manuscript draft. JV performed the mass spectrometric and chemical analysis and revised the manuscript. CR carried out the genome sequencing and assembling. XHC participated in experimental design and revised the manuscript. JB provided genome sequence database support. ML performed the SEM observation. JJR participated in the manual annotation of the genome sequence. QW guided experimental design. RB guided experimental design, performed data analysis and annotation and wrote the final version of the manuscript. All authors read and approved the final manuscript.
